# Field survey and molecular characterization of apicomplexan parasites in small mammals from military camps in Afghanistan

**DOI:** 10.1007/s00436-023-07820-8

**Published:** 2023-03-22

**Authors:** Ulrich Schotte, Alfred Binder, Katja V. Goller, Michael Faulde, Silke Ruhl, Sabine Sauer, Mathias Schlegel, Jens P. Teifke, Rainer G. Ulrich, Claudia Wylezich

**Affiliations:** 1 Department of Veterinary Medicine, Central Institute of the Bundeswehr Medical Service Kiel, Kopperpahler Allee 120, 24119 Kronshagen, Germany; 2 Institute of Diagnostic Virology, Friedrich-Loeffler-Institut, Federal Research Institute for Animal Health, Südufer 10, 17493 Greifswald Insel Riems, Germany; 3 Institute for Hygiene and Environmental Medicine and Central Unit for Infection Prevention and Control, University Medicine Greifswald, Fleischmannstraße 8, 17475 Greifswald, Germany; 4 Department of Medicine, Central Institute of the Bundeswehr Medical Service Koblenz, Andernacher Str. 100, 56070 Koblenz, Germany; 5 Bundeswehr Research Institute (WIWeB), Institutsweg 1, 85435 Erding, Germany; 6 Bundeswehr Medical Academy, Deployment Health Surveillance Center, Neuherbergstr. 11, 80937 Munich, Germany; 7 Division E, Bundeswehr Medical Academy, Military Medical Research and Development, Neuherbergstr. 11, 80937 Munich, Germany; 8 Institute of Novel and Emerging Infectious Diseases, Friedrich-Loeffler-Institut, Federal Research Institute for Animal Health, Südufer 10, 17493 Greifswald Insel Riems, Germany; 9 Seramun Diagnostica GmbH, Spreenhagener Str. 1, 15754 Heidesee, Germany; 10 Department of Experimental Animal Facilities and Biorisk Management (ATB), Friedrich-Loeffler-Institut, Federal Research Institute for Animal Health, Südufer 10, 17493 Greifswald Insel Riems, Germany

**Keywords:** Rodent, Afghanistan, *Hepatozoon*, *Klossiella*, Molecular phylogeny, Shrew

## Abstract

Small mammals are an important reservoir for causative agents of numerous infectious diseases, including zoonotic and vector-borne diseases. The occurrence of these pathogens represents a regional but permanent threat for humans and animals in general and might especially weaken military personnel and companion animals in abroad missions. In our study, small mammals collected in military camps in Afghanistan (Feyzabad, Mazar-e Sharif, and Kunduz) were investigated for the presence of apicomplexans using histopathology and molecular methods. For this purpose, well-established and newly developed real-time PCR assays were applied. A high prevalence was detected not only in house mice (*Mus musculus*), but also in shrews (*Crocidura* cf. *suaveolens*) and grey dwarf hamsters (*Cricetulus migratorius*). The molecular characterization based on the 18S rRNA gene revealed a close relationship to a cluster of *Hepatozoon* sp. detected in voles of the genus *Microtus. Hepatozoon canis* DNA was detected in one house mouse as well as in two *Rhipicephalus* ticks from a dog puppy. In addition, around 5% of the house mice were found to be infected with far related adeleorinids showing the highest sequence identity of 91.5% to *Klossiella equi*, the only published *Klossiella* sequence at present. For their better phylogenetic characterization, we conducted metagenomics by sequencing of two selected samples. The resulting 18S rRNA gene sequences have a length of about 2400 base pairs including an insertion of about 500 base pairs and are 100% identical to each other. Histopathology together with organ tropism and detection rates verified this sequence as of *Klossiella muris*. In conclusion, we documented naturally occurring protozoan stages and the additional taxonomic characterization of a well-known commensal in mice by applying a combination of different approaches. The study is of medical, social, and biological importance for ensuring human and animal health in military camps and also stresses the required awareness for the potential risk of zoonoses.

**Supplementary Information:**

The online version contains supplementary material available at 10.1007/s00436-023-07820-8.

## Introduction

Infectious diseases are of major concern in military operations and through the ages, wartime epidemics severely weakened the military capabilities (Johnson 2001). In case of military operations abroad, soldiers often have to face infectious diseases, for which they are initially fully vulnerable. Due to available vaccinations and high hygienic and preventive measures, the risk of epidemics in deployed troops can be reduced drastically (Murray et al. 2007). However, vector-borne diseases represent a regional but permanent risk, especially in the absence of specific and efficient prophylaxis (Pages et al. 2010). Besides vector monitoring and control, the identification of possible reservoirs gives additional information on circulating infectious agents, which allows a more comprehensive approach for the implementation of effective preventive measures. This is not only important for deployed military personnel but also for military dogs, which became an important part in military operations not only for the detection of explosives or mines but also as military service or therapy service dogs.

Small mammals like rodents and shrews serve as reservoirs for causative agents of numerous infectious and zoonotic diseases. Based on present reservoir species and vectors, (sub)regional infectious cycles exist and often pose a serious health threat for people as well as companion animals living in these regions (Davis et al. 2005; Meerburg et al. 2009; Yabsley and Shock 2013; Dahmana et al. 2020). Among such zoonotic pathogens are viruses, such as bunya-, flavi-, and filoviruses, bacteria such as *Leptospira* spp., but also endoparasites including apicomplexans like *Babesia microti* (Karbowiak 2004, Zeng et al. 2022, Eisen 2023). Some apicomplexan genera like *Babesia *or *Hepatozoon *are also well known for their significance in canine health, where small mammals may serve as important reservoirs (Johnson et al. 2009; Demoner et al. 2016).

Adeleorinid coccidia (Apicomplexa) in general include not only heteroxenous parasite families like Hepatozoidae, Dactylosomatidae, Hemogregarinidae, and Karyolysidae but also monoxenous parasites of the families Adeleidae, Legerellidae, and Klossiellidae (Barta et al. 2012; O'Donoghue 2017). These coccidia infect multiple vertebrate as well as invertebrate hosts and are characterized by a complex life cycle involving one or more asexual cycles of merogony, syngamy, and sporogony in their intermediate hosts followed by gametogony, syngamy, and sporogony in their definitive hosts (Barta et al. 2012). Except some more prominent apicomplexans like *Babesia* spp. and *Hepatozoon* spp., for that targeted molecular characterization is possible, the taxonomic classification of other apicomplexans is usually based on morphological characters and biological features including the respective developmental stages in different hosts. Due to slowly increasing molecular data of some parasite taxa, although described since decades, sometimes sequences still do not allow a precise taxonomic classification.

The genus *Hepatozoon* (Adeleorina, Coccidia, Apicomplexa) comprises more than 100 species, which infect a wide range of vertebrate hosts from amphibians and reptiles to birds and mammals (Smith 1996; Merino et al. 2014; O'Donoghue 2017). Their basic life cycle includes the sporogonic development and oocyst formation in the hematophagous invertebrate as the final host, whereas merogony and gamontogonic development occurs in the vertebrate intermediate host. Vertebrate hosts get infected by ingestion of the invertebrate hosts as described for *H*. *catesbianae* in bull frogs (Smith 1996) or *H*. *canis* and *H. americanum* in dogs (Vincent-Johnson 2003; Baneth 2011). For many *Hepatozoon* species infecting snakes, a prey-predator infectious life cycle is accepted with additional paratenic vertebrate hosts like rodents beside lizards or frogs (Smith 1996; Sloboda et al. 2008; Tome et al. 2014). The prey-predator life cycle is also described in some wild canid and rodent species (Maia et al. 2014b). Besides these naturally occurring life cycles, often without clinical relevance, the infection of dogs with *H. canis* or *H. americanum* has health consequences varying from inapparent, sometimes mild to severe clinical outcome, and occasionally death (Baneth et al. 2003).

The genus *Klossiella* (Adeleorina, Coccidia, Apicomplexa) comprises less than 20 species. Most of them infect the renal tract of a wide variety of mammals, whereas one species infects the intestinal tract of snakes. Gametogony and sporogony typically occur in the kidney in which the infection rarely results in an inflammation of the renal tissue (Taylor et al. 1979; Leveille et al. 2019). Thus, the genus is considered apathogenic.

The study focused on the prevalence of zoonotic but also dog-related parasites in small mammals inside military camps during the International Security Assistance Force operation (Schlegel et al. 2012a). Therefore, rodents and shrews (Gertler et al. 2017) and a few ticks from Northern Afghanistan were collected and investigated to estimate the prevalence of apicomplexan taxa in order to identify potential sources for zoonotic and canine infectious diseases inside military camps and to broaden the knowledge on occurring species from a yet hardly studied region.

## Material and methods

### Collection of small mammals and ticks in Afghanistan

In three German military camps located near Mazar-e Sharif (MES), Kunduz (KDZ), and Feyzabad (FEY) in Northern Afghanistan, 751 rodents and other small mammals were collected as part of army pest control measures from January 2009 to November 2011. The present study comprised 388 house mice (*Mus musculus*), 42 grey dwarf hamsters (*Cricetulus migratorius*), eight lesser white-toothed shrews (*Crocidura* cf. *suaveolens*), one *Suncus* shrew (*Suncus* sp.), and one Asian house rat (*Rattus tanezumi*), mainly collected from 2009 to 2010 (*n* = 284, and *n* = 105, respectively), with some additional animals trapped in 2011 (*n* = 29). The small mammals originated from MES (*n* = 344), KDZ (*n* = 62), and FEY (*n* = 27). For 22 animals, no exact sampling date, and for seven animals, the trapping site information was missing (Gertler et al. 2017). We additionally included here also 53 ticks (mostly *Rhipicephalus* sp., seven adults, 46 nymphs) taken from an approximately 6 months old puppy stray dog in MES in May 2011.

### Necropsy, sampling, and histopathology

For all small mammals, complete post mortem examinations were conducted according to a standard protocol in a biosafety level 3 containment necropsy suite (Gertler et al. 2017). The phenotypical identification of small mammal species was accompanied by photo documentation of each animal, documentation of gross pathology, and subsequent *cytochrome b*-based molecular species confirmation (Schlegel et al. 2012b; Gertler et al. 2017). Tissues were collected in specific order as described before (Ulrich et al. 2011; Schlegel et al. 2012a), placed in 1.5-ml reaction tubes and stored at − 20 °C until further analysis. For the molecular detection of apicomplexan taxa, kidney, liver, and spleen were selected if available. Not all tissues were available for histopathologic evaluation. However, representative tissue samples from the major organs were collected for histopathology including in most cases brain, heart, and skeletal muscle, lung, liver, kidney, spleen, gut, and haired skin. The tissues were fixed in 10% neutral buffered formalin and routinely processed and stained with hematoxylin and eosin (HE). Microscopic analysis was performed by an ACVP (American College of Veterinary Pathologists) board–certified pathologist (JPT).

### DNA extraction and PCR screening from kidney, liver, and spleen samples

Approximately 25 mg of each organ sample was homogenized in a TissueLyser (Qiagen, Hilden, Germany) and lysed through the addition of ATL buffer (Qiagen) and proteinase K (Qiagen) for 5 min at 56 °C. DNA extraction was performed using the QIAamp DNA Mini Kit (Qiagen) following the instructions of the manufacturer. The final DNA purity and concentration of each sample was measured using a NanoDrop spectrophotometer (Thermo Scientific, Wilmington, MA, USA).

The whole sample panel was initially screened for apicomplexan 18S ribosomal RNA (rRNA) gene sequences targeting the 3′ region via real time PCR (qPCR, Table 1) according to Rembeck (2006). From each sample, 3 µl of DNA was added to a 22-µl reaction mixture (QuantiTect Probe PCR Kit, Qiagen) with 1.2 mM of each primer and 0.016 mM probe. The amplification was performed in a Mx3005P thermal cycler (Stratagene/Agilent, Santa Barbara, CA, USA) using the following parameters: 94 °C for 20 min, followed by 50 cycles of 94 °C for 30 s, 50 °C for 30 s, and 72 °C for 30 s. From each animal, all available DNA samples extracted from kidney (*n* = 440), liver (*n* = 43), and spleen (*n* = 301) were screened. Samples with a cycle quantification (cq)-value < 40 were classified as positive, samples with cq-values ≥ 40 after repeated testing were classified as questionable, and samples with no cq-value were classified as negative.

**Table 1:** Primers and probes used in this study. All primer pairs are targeting regions of the 18S rRNA

Designation	Sequence 5′-3′	Reference
PIR2	5′-CgA ATA ATT CAC Cgg ATC AC-3′	Rembeck (2006)
PIR3	5′-AAT CAT gAA CgA ggA ATg C-3′	
PIR-S-2	5′-**FAM**-CgG gAT ACA CAC CgC CCg TCg CT TC gCg-**BBQ**-3′	
BabR	5′-gTg AAA CTg CgA ATg gCT CA-3′	Inokuma et al. (2003)
BabF	5′-CCA TgC TgA AgT ATT CAA gAC-3′	
HepF	5′-ATA CAT gAg CAA AAT CTC AAC-3′	Inokuma et al. (2002)
HepR	5′-CTT ATT ATT CCA TgC TgC Ag-3′	
Adel1_F	5′-CAA TTC TAA CAg CAT AAg AgA-3′	This study
Adel1_R	5′-CTA TCA TTC CAA TTA CAA AgC-3′	
Adel1_probe	5′-**HEX**-gTg ACA AgA AAT AAC AgT ACA Agg CAg–**BHQ2**-3′	
Adel2_F	5′-TTT CTg CCg AAg gCg ACT g–3′	
Adel2_R	5′-AgC AgT TAA gCT CCg gAA ATC–3′	
Adel2_probe	5′-**FAM**-gTA AgA gTA gTA TCT TAg TgC gCT TTC–**BHQ1**-3′	

### Sanger sequencing and cluster-specific qPCR

For Sanger sequencing, samples tested positive for apicomplexan DNA were retested by conventional PCR targeting the 5′ region of the 18S rRNA gene of *Babesia* sp. (BabRF-PCR, according to Inokuma et al. 2003) or *Hepatozoon* sp. (HepRF-PCR, according to Inokuma et al. 2002) revealing PCR products of 650 base pairs (bp) and 780 bp, respectively. Sequencing of the PCR products after purification was performed in both directions by SeqLab (Göttingen, Germany) using the respective PCR primers. Obtained sequences were compared to publicly available sequences in GenBank, and a sequence analysis by multiple sequence alignment based on ClustalW-algorithm in Bioedit 7.1.7 and phylogenetic tree reconstruction using Mega 5.2 (Tamura et al. 2011) was performed. Based on the sequencing results, two qualitative cluster-specific qPCR assays targeting the 5′ region of the 18S rRNA gene were designed. The adeleorinid cluster 1-specific qPCR (“Adel1,” Table 1) amplifies a region of 82 bp, and the adeleorinid cluster 2-specific assay (“Adel2,” Table 1) generates a 118-bp product. These assays were applied with 5 µl of DNA using the aforementioned kit (QuantiTect Probe PCR Kit, Qiagen) and composition with the following parameters: 94 °C for 20 min, followed by 40 cycles of 94 °C for 30 s, 54 °C (adeleorinid cluster 1 qPCR) or 58 °C (adeleorinid cluster 2 qPCR) for 30 s, and 72 °C for 30 s. Samples with a cq-value < 40 were classified as positive, samples with cq-values ≥ 40 after repeated testing were classified as questionable, and samples with no cq-value were classified as negative.

### Statistical analysis

Small mammals were classified positive, when one to three tested tissue samples per animal were positive by one PCR assay. All analyses were done using EpiTools epidemiological calculators (Sergeant 2018). Prevalence per species and location and their 95% confidence intervals (CIs) were calculated using “Calculate confidence limits for a sample proportion” (https://epitools.ausvet.com.au/ciproportion). Initial testing for statistical significance was carried out with a chi-square test (http://epitools.ausvet.com.au/content.php?page=chi_sq). In the case of significance, the comparison of two prevalence values was further tested with the 2-sample t-test for summary data (http://epitools.ausvet.com.au/content.php?page=2-sample-t-test).

### RNA extraction, high-throughput sequencing, and sequence analysis

Selected samples were subjected to high-throughput sequencing (HTS) to obtain more sequence information of the apicomplexan parasites of adeleorinid cluster 2, namely KS/11/1479 (library L3852) and KS/11/1691 (library L3853). RNA was extracted after sample disintegration using the cryoPREP impactor (Covaris, Brighton, UK) and sequencing libraries were constructed as detailed described (Wylezich et al. 2018) and sequenced using Ion Torrent S5XL instrument. The Genome Sequencer software suite (versions 2.6; Roche) was used to perform a pre-mapping analysis with *Klossiella equi* MH211602 (Leveille et al. 2019) as reference sequence as described (Wylezich et al. 2019). Obtained *Klossiella* 18S rRNA sequence fragments were used to refine the mapping analysis applying an identity threshold of 99% and minimum overlap length of reads of 99%. Sequences were improved and prolonged using an iterative mapping and assembly process using the abovementioned software tool. Underlying sequence reads of the final sequences were visualized with Geneious Prime 10.2.3 (Biomatters, Auckland, New Zealand; https://www.geneious.com) and inspected manually. The variant analysis tool integrated in Geneious Prime 10.2.3 was applied to identify potential single nucleotide variants (SNVs) and the acquisition of variant frequencies for each variant (default settings, minimum variant frequency 0.02).

### Phylogenetic reconstruction using ribosomal RNA gene sequences

Alignments of sequences obtained in this study and sequences retrieved from GenBank were performed using MAFFT version 7.388 (Katoh and Standley 2013) as implemented in Geneious Prime 10.2.3. Two different alignment datasets were prepared: (i) an alignment with partial 18S rRNA gene sequences including the apicomplexan sequences obtained by Sanger sequencing in this study; (ii) an alignment for *Klossiella* 18S rRNA gene sequences obtained by HTS in this study including nearly complete 18S rRNA gene sequences. Phylogenetic trees were reconstructed using PhyML version 3.0 (Guindon et al. 2010) using the GTR + GAMMA + I model with 1000 bootstrap replications and MrBayes version 3.2.6 (Ronquist and Huelsenbeck 2003) using the GTR model with eight rate categories and a proportion of invariable sites within the Geneious Prime 10.2.3 software package. The Bayesian analysis was performed for 10^6^ generations and sampled every 1000 generations for four simultaneous chains.

## Results

### Initial qPCR screening of small mammals for apicomplexans

Renal eluates from 440 small mammals and spleen sample eluates from 319 small mammals were initially screened by qPCR as described by Rembeck (2006) for apicomplexan infections. The screening revealed a total of 73 of 388 house mice (18.8%, CI 95% ± 3.9%) qPCR positive in one or both samples from all three military camps with remarkable prevalence differences between the three sites (Table 2). In detail, 53 out of 305 screened house mice (*Mus musculus*) from MES (17.4%, CI ± 4.3%), 18 out of 61 from KDZ (29.5%, CI ± 11.5%), and 2 out of 18 from FEY (11.1%, CI ± 14.5%) were initially qPCR-positive. In addition, two of eight *Crocidura* cf. *suaveolens* shrews were found to be qPCR-positive, both animals originated from MES. None of the other animals (*n* = 42 grey dwarf hamsters, *n* = 1 *Suncus* shrew, and *n* = 1 rat) were qPCR-positive. In addition, two out of seven adult female ticks were tested positive, one *Rhipicephalus* sp., and one tick that could not be systematically determined. All 46 nymphs were tested negative.

**Table 2:** Apicomplexan screening of rodents and other small mammals from German military camps in Mazar-e Sharif (MES), Kunduz (KDZ), and Feyzabad (FEY) in Afghanistan by qPCR assay according to Rembeck (2006)

	Animals tested [*n*]/tested positive/% ± CI 0.95 per site				
Species	MES	KDZ	FEY	n/a	Total
House mouse (*Mus musculus*)	305/53/ 17.4% ± 4.3%	61/18/ 29.5% ± 11.5%	18/2/ 11.1% ± 14.5%	4/0/–	388/73/ 18.8% ± 3.9%
Grey dwarf hamster (*Cricetulus migratorius*)	30/0/–	–	9/0/–	3/0/–	42/0/–
Lesser white-toothed shrew (*Crocidura* cf. *suaveolens*)	7/2/ 28.6% ± 33.5%	1/0/–	–	–	8/2/ 25.0% ± 30.0%
*Suncus* shrew (*Suncus* sp.)	1/0/–	–	–	–	1/0/–
Asian house rat (*Rattus tanezumi*)	1/0/–	–	–	–	1/0/–
total	344/55/ 16.0% ± 3.9%	62/18/ 29.0% ± 11.3%	27/2/ 7.4% ± 9.9%	7/0/–	440/75/ 17.1% ± 3.5%

*n/a* sampling site information not available

### Phylogenetic analysis of detected taxa

For further characterization, 48 from initially 72 qPCR-positive small mammals with cq-values below 35 were selected for further investigation by two conventional PCR assays and subsequent sequence determination and phylogenetic analysis. First, *Babesia* sp.-targeting BabRF-PCR according to Inokuma et al. (2003) revealed evaluable results for eight animals. Further testing with the HepRF-PCR (Inokuma et al. 2002) resulted in evaluable results from another 10 animals. Three animals gave evaluable results in both PCR assays (KS/11/1555, KS/11/1687, and KS/11/1763). In total, sequences from 18 rodents and two from ticks were used for subsequent sequence analysis (Supplementary Tables S1 and S2).

**Fig. 1:**
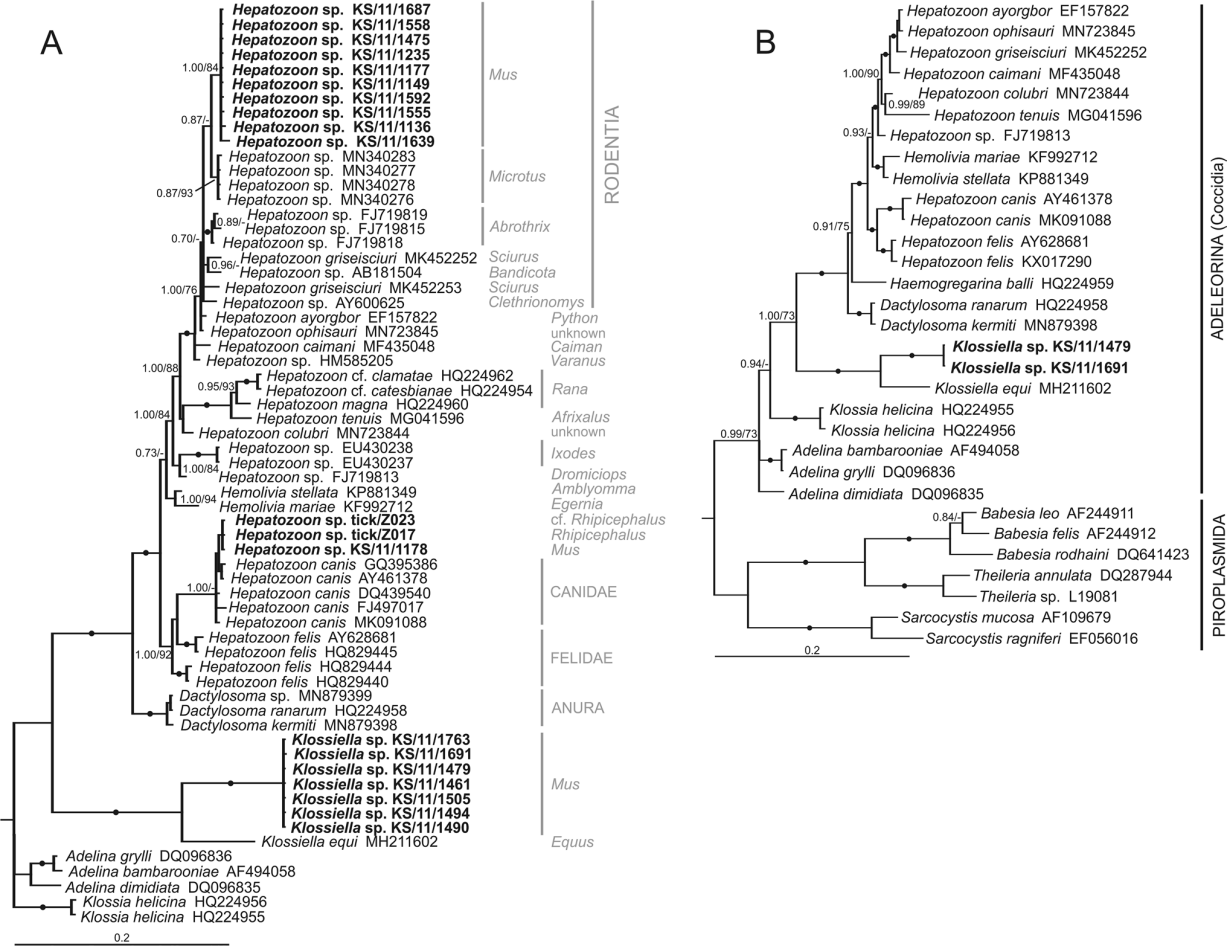
Phylogenetic tree of partial 18S rRNA gene sequences including the apicomplexan sequences obtained by Sanger sequencing (**A**). Information on host taxonomy is given in grey letters. The tree was rooted with the outgroup taxon *Sarcocystis* species (AF109679, EF056016, not shown). **B** Phylogenetic tree of *Klossiella* 18S rRNA gene sequences obtained by HTS including nearly complete 18S rRNA gene sequences. The tree was rooted with the outgroup taxon *Cryptosporidium* species (AF108862, AF108864, not shown). Sequences obtained in this study are written in bold letters. Support values (MrBayes/Maximum Likelihood) above 70% are given; dots represent values of 1.00/100

Obtained sequences branched off in three major clusters (Fig. 1A): sequences from 10 house mice clustered close to *Hepatozoon* sp. detected in *Microtus* sp. (> 98.9–99.3% sequence identity, Fig. 1A). One additional sample from a house mouse trapped in MES as well as samples from two ticks clustered within the *Hepatozoon canis* clade (> 99% sequence identity). Sequences from another seven house mice branched with *Klossiella equi* detected in *Equus ferus caballus* (about 91% sequence identity).

HTS enabled the determination of two full-length 18S rRNA gene sequences, verifying animals positive for adeleorinid cluster 2 qPCR to be infected with *Klossiella* sp. based on sequence similarity with the sequence from *Klossiella equi* (Leveille et al. 2019) (Fig. 1B). Both *Mus*-derived sequences showed a 512-bp insertion located in the V7 region of the 18S rRNA gene expanding its length to about 2400 bp. The consensus sequences are 100% identical to each other with only one TA transversion with T in KS/11/1691 (L3853) at position 777. This is a SNV in KS/11/1479 (L3852, position 760) with a variant frequency of 63% A (53% strand bias). A minor SNV was detected in KS/11/1479 (L3852) at position 1265, a CT transition with 19% frequency (63% strand bias). The insertion had a lower GC content (37%) as the complete gene (41%) and consisted of repetitive and low-complexity sequences (Supplementary Fig. S1). Infections with other apicomplexans, like piroplasms (*Babesia* spp.), were not detected in any sample.

The nucleotide sequence data reported in this study are available in GenBank under the NCBI accession numbers MT664753–MT664772.

### Re-evaluation of the prevalence with cluster-specific qPCR assays

**Fig. 2:**
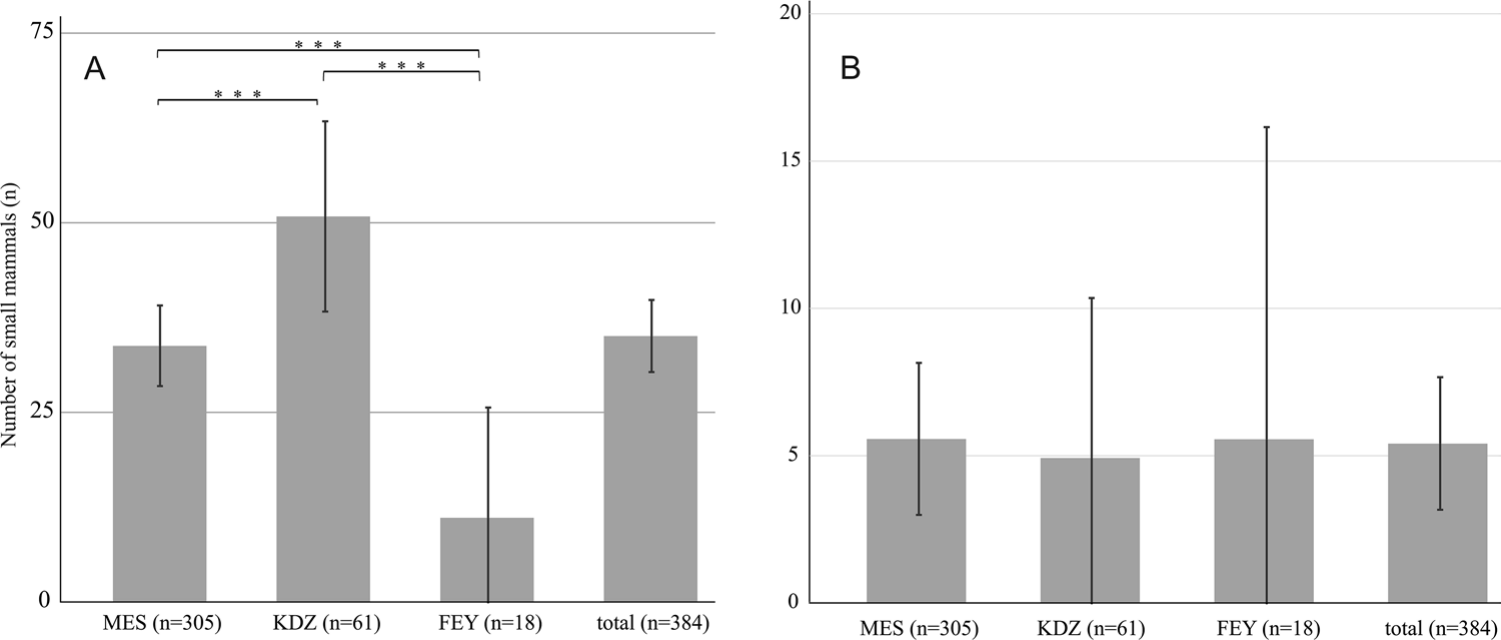
Prevalence of *Hepatozoon* sp. (adeleorinid cluster 1, A) and of *Klossiella* sp. (adeleorinid cluster 2, B) in house mice (*Mus musculus*) from three different German military camps in MES-Mazar-e Sharif, KDZ-Kunduz, and FEY-Feyzabad in Afghanistan collected during 2009 to 2011. Cq-values with upper and lower CI95%; significant differences are indicated. The sample proportions were tested for significance by 2-sample *t*-test for summary data. *** indicates a *p*-value < 0.001

To evaluate the prevalence of apicomplexan parasites within the small mammal populations, we designed two specific qPCR assays Adel1 (*Hepatozoon*-specific) and Adel2 (*Klossiella*-specific, Table 1) for the qualitative detection of the identified taxa. Retesting of eluates from 440 animals with the Adel1 assay revealed positive samples of house mice, grey dwarf hamsters, and *Crocidura* cf. *suaveolens* shrews and a mean prevalence of 35.1%, CI 95% ± 4.8% (Table 3). *Hepatozoon* (cluster 1)-positive house mice were identified at all sampling sites, revealing a significantly different sampling location-dependent prevalence in mice between 11.1% (CI95% ± 12.6%) and 50.8% (CI95% ± 12.6%; mean 35.1% CI95% ± 4.8%; Table 3, Fig. 2(A). The trapped *Suncus* shrew and the Asian house rat (one each) were negative for *Hepatozoon* sp.

**Table 3:** Prevalence of *Hepatozoon* sp. (adeleorinid cluster 1) in small mammals from German military camps in Mazar-e Sharif (MES), Kunduz (KDZ), and Feyzabad (FEY) in Afghanistan determined by the newly designed adeleorinid cluster 1-specific qPCR (“Adel1”) assay

	Animals tested [*n*]/tested positive/% ± CI 0.95 per site				
Species	MES	KDZ	FEY	n/a	Total
House mouse (*Mus musculus*)	305/103/ 33.8% ± 5.3%	61/31/ 50.8% ± 12.6%	18/2/ 11.1% ± 12.6%	4/0/–	388/136/ 35.1% ± 4.8%
Grey dwarf hamster (*Cricetulus migratorius*)	30/1/ 3.3% ± 6.4%	–	9/1/ 11.1% ± 20.5%	3/0/–	42/2/ 4.8% ± 6.4%
Lesser white-toothed shrew (*Crocidura* cf. suaveolens)	7/3/ 42.9% ± 36.7%	1/0/–	–	–	8/3/ 37.5% ± 33.6%
*Suncus* shrew (*Suncus* sp.)	1/0/–	–	–	–	1/0/–
Asian house rat (*Rattus tanezumi*)	1/0/–	–	–	–	1/0/–
Total	344/107/ 31.1% ± 4.9%	62/31/ 50.0% ± 12.5%	27/3/ 11.1% ± 11.9%	7/0/–	440/141/ 32.1% ± 4.4%

Adel2 (*Klossiella/*cluster 2) assay positive samples were detected in house mice from all sampling sites. *Klossiella* prevalences for mice were at comparable low detection rates between 4.9% (CI95% ± 5.4%) and 5.6% (CI95% ± 10.6%; mean 5.4% (CI95% ± 2.2%; Table 4) without significant differences among sampling sites (Fig. 2(B). The other animals (*n* = 42 grey dwarf hamsters, *n* = 8 *Crocidura* cf. *suaveolens* shrews, *n* = 1 Suncus shrew, and *n* = 1 rat) were qPCR-negative for *Klossiella*/cluster 2.

**Table 4:** Prevalence of *Klossiella* sp. (adeleorinid cluster 2) in small mammals from German military camps in Mazar-e Sharif (MES), Kunduz (KDZ), and Feyzabad (FEY) in Afghanistan determined by the newly designed adeleorinid cluster 2-specific qPCR (“Adel2”) assay

	Animals tested [*n*]/tested positive/% ± CI 0.95 per site				
Species	MES	KDZ	FEY	n/a	Total
House mouse (*Mus musculus*)	305/17/ 5.6% ± 2.6%	61/3/ 4.9% ± 5.4%	18/1/ 5.6% ± 10.6%	4/0/–	388/21/ 5.4% ± 2.3%
Grey dwarf hamster (*Cricetulus migratorius*)	30/0/–	–	9/0/–	3/0/–	42/0/–
Lesser white-toothed shrew (*Crocidura* cf. *suaveolens*)	7/0/–	1/0/–	–	–	8/0/–
*Suncus* shrew (*Suncus* sp.)	1/0/–	–	–	–	1/0/–
Asian house rat (*Rattus tanezumi*)	1/0/–	–	–	–	–
Total	344/17/ 4.9% ± 2.3%	62/3/ 4.8% ± 5.3%	27/1/ 3.7% ± 7.1%	7/0/–	440/21/ 4.8% ± 1.9%

Samples from five house mice were positive in both assays (Supplementary Tables S1 and S2). The trapped *Suncus* shrew and the Asian house rat (one each) were negative in both cluster-specific assays.

### Organ distribution, and histopathology

Initially, the organ distribution of *Hepatozoon*- and *Klossiella*-related apicomplexans was estimated. All investigated organs gave positive qPCR results for both taxa, although at different detection rates. This demonstrates the wide systemic infestation of the investigated small mammals from Afghanistan with *Hepatozoon* and *Klossiella*.

**Fig. 3:**
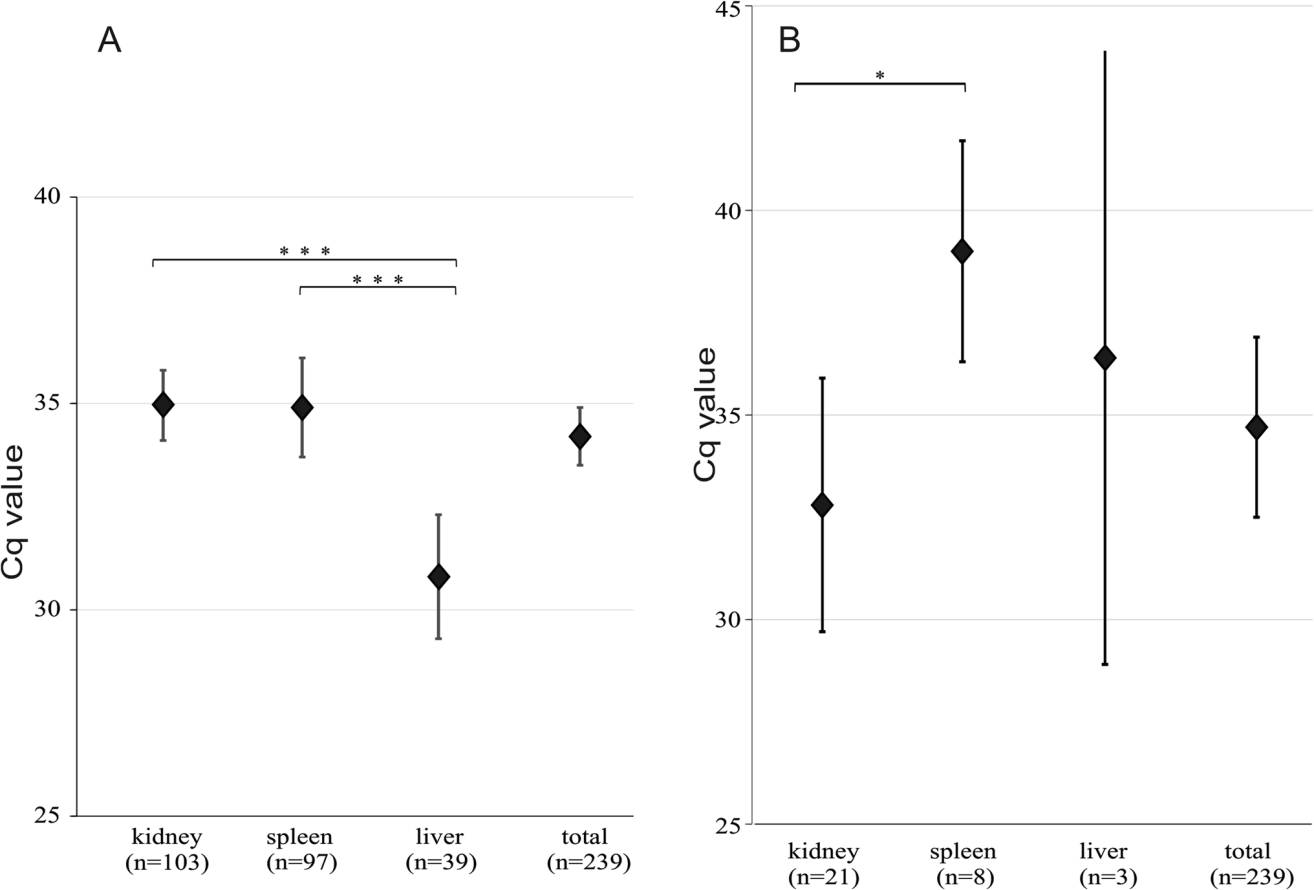
Mean cq-values per tissue sample after detection of *Hepatozoon* spp. (adeleorinid cluster 1, **A**) in small mammals and *Klossiella* sp. (adeleorinid cluster 2, **B**) in house mice (*Mus musculus*) from German military camps in MES-Mazar-e Sharif, KDZ-Kunduz, and FEY-Feyzabad in Afghanistan collected during 2009 to 2011. Cq-values with upper and lower CI 95%; significant differences are indicated. The sample proportions were tested for significance by 2-sample *t*-test for summary data. *** indicates a *p*-value < 0.001, * indicates low significance with a *p*-value < 0.05

For *Hepatozoon* sp.-positive animals, all liver samples had a significantly lower cq-value than samples from kidney (*t* = 4.644, *p* < 0.001) and spleen (*t* = 3.736, *p* < 0.001; Fig. 3A). In contrast, *Klossiella*-positive kidney samples had significantly lower cq-values compared to spleen samples (*t* = 2.48, *p* < 0.05), and a lower mean cq-value than liver samples although not statistically significant due to small sample size (Fig. 3B).

**Fig. 4:**
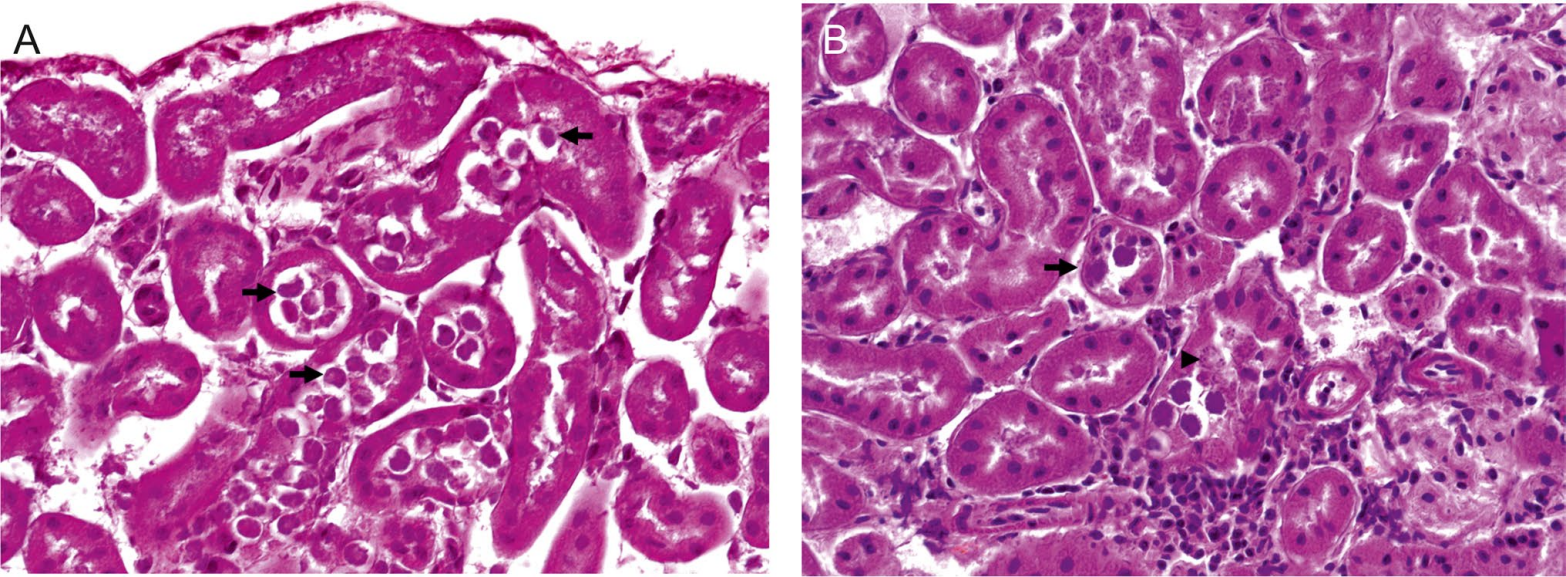
Tubular epithelium containing various developmental stages of *Klossiella* spp. (arrows). H&E stain. **A** KS/11/1479, house mouse, MES, AFG and **B** KS/11/1691, house mouse, MES, AFG

In six from 21 house mice tested positive for *Klossiella* DNA, protozoan structures corresponding in morphology to *Klossiella muris* (Smith and Johnson 1902) were documented in slides after HE staining (Fig. 4). Kidneys from three additional house mice showed interstitial nephritis similar to the pathomorphology seen from animals infected with *Klossiella muris* (Supplementary Table S1). Both sporogenic and gametogenic stages of *K.* *muris* were identified in the individual tubular epithelial cells (Fig. 4). Schizonts were mostly present in the proximal convoluted tubules, but also free within the lumen of the tubules (Fig. 4A). Budding sporonts (arrowhead Fig. 4B) and all developmental stages of sporoblasts (arrow Fig. 4A) were observed protruding into the tubular lumen. Sporocysts were rupturing out of the mature sporoblast into the lumen of tubules. Renal tubules were partly dilated and contained cellular debris. Focally, intratubular stages of *Klossiella muris* were associated with multifocal mild tubular necrosis with focal interstitial infiltrates of lymphocytes and plasma cells. No further lesions were detected by histopathologic investigation correlating with the results from cluster-specific PCR testing, including the lack of findings correlating with *Hepatozoon* infection.

## Discussion

Rodents are worldwide well known as occurring reservoirs for multiple pathogens, though the occurrence of certain rodent species and their infectious passengers varies with region, and habitat, as well as general ecological circumstances. Parallel studies focusing on bacterial and viral zoonotic pathogens in small mammals, mainly house mice, from military camps in Northern Afghanistan revealed at most low detection rates for numerous zoonotic pathogens (Mayer-Scholl et al. 2019; Essbauer et al. 2022) and non-zoonotic agents (Gertler et al. 2017). In our study with focus on vector-borne protozoans in this small mammal sample set, we were able to show the occurrence of two apicomplexan genera in rodents and shrews with notable detection rates in different organs. Hence, due to the low number of grey dwarf hamsters, shrews, and one rat available for this investigation, these data should be taken with caution. We could not fully exclude the presence of some apicomplexan parasites in these animal species when not detected.

*Hepatozoon* species have been detected in different small rodent populations from various regions worldwide (Smith 1996). Some rodent species harbor *Hepatozoon* species, which to date are more restricted to their host species like *Hepatozoon erhardovae* frequently detected in bank voles (*Myodes* [*Clethrionomys*] *glareolus* [Schreber]) (Walter and Liebisch 1980; Healing 1981; Laakkonen et al. 2001), *Hepatozoon sylvatici* in wood mice *Apodemus sylvaticus* and yellow-necked field mice *A. flavicollis* (Frank 1977; Walter and Liebisch 1980), and *Hepatozoon* sp. in field voles *Microtus agrestis* (Healing 1981). The detection of a clinical hepatozoonosis in a multimammate mouse (*Mastomys natalensis*) from Uganda was classified as a rather accidental infection (Krampitz et al. 1968). In susceptible hosts, high detection rates between 30 and > 70% are reported. In our study, we detected *Hepatozoon* in more than 30% of tested house mice, leading to the assumption that a persistent infectious cycle exists with house mice involved as intermediate hosts in Afghanistan. In addition, grey dwarf hamsters and *Crocidura* shrew species at least may serve as accidental host, demonstrating the wide host range of *Hepatozoon* in nature. Moreover, the classification of mice solely as a paratenic host should be discussed. Our *Hepatozoon*-sequences from Afghanistan cluster with high sequence similarity within the rodent associated *Hepatozoon* cluster usually part of a predator–prey life cycle (Sloboda et al. 2008; O'Donoghue 2017). It is, however, questionable if the buildup of military building, thus serving as suitable artificial ecotopes for commensal mammals, resulted in a shift within the small mammal population inside the camp leading to different dominant rodents like house mice (Gertler et al. 2017). Recently published data from Northern China describe *Hepatozoon ayorgbor*-like sequences detected in great gerbils (*Rhombomys opimus*), although with distinct lower detection rates (Ji et al. 2021). Presuming that house mice are more susceptible to naturally occurring *Hepatozoon* sp., a specific food availability driven high population density of house mice may further facilitate higher infection rates. Hence, due to the lack of samples from snakes or possible vectors from the respective regions, the final verification of a prey-predator life cycle is not possible.

The standard method for the detection of *Hepatozoon* sp. is the microscopical observation of parasitic stages within lymphocytes on Wright and Giemsa stains in thin blood smears of tissue sections (Baneth 2013). However, although successful in acute cases with high parasite loads, the microscopic detection lacks sensitivity when trying to detect chronic cases or the nonclinical infection of reservoir hosts. Moreover, a diagnosis solely based on morphological characteristics hampers the differentiation of closely related species (Wylezich et al. 2020). In addition, the morphological characteristics of the same species may vary when different hosts are infected (Demeter et al. 2011). Therefore, the sensitive and specific detection using molecular methods becomes more and more widespread in recent studies although it lacks relation to former studies describing *Hepatozoon* sp. from rodents. In our study, we were able not only to verify the presence of different *Hepatozoon* species in rodents from Afghanistan by sequence analysis, but also to estimate the prevalence using newly designed sensitive qPCR assays. Although we were able to confirm the occurrence of *Hepatozoon* in mice when employing PCR and sequencing techniques, the histological detection failed. This is in line with the findings of Sloboda et al. (2008), who could not detect life stages of *H. ayorgbor* in infected mice even under experimental conditions, although these mice were tested positive by PCR and were able to infect snakes. Our results thus confirm former experimental findings under field conditions that mice can play an important role in the life cycle of *Hepatozoon* species. The remarkable higher sensitivity of qPCR assays compared to conventional PCR and microscopy was also previously demonstrated based on lizards collected in Portugal (Maia et al. 2014a).

The detection of *Hepatozoon canis* in one house mouse, although first time reported from a natural environment, should be interpreted as accidental infection due to the presence of *Hepatozoon canis* in this region as verified by the additional detection in ticks collected from a stray dog inside the camp. The proof of susceptibility of house mice to *Hepatozoon canis* failed under experimental circumstances (Nordgren and Craig 1984) as well as the attempted detection during a field survey of rodents for American canine hepatozoonosis (Johnson et al. 2007). The molecular detection of *Hepatozoon americanum* after experimental transmission but without evidence of visible infectious stages is in line with our findings (Johnson et al. 2008) and leads to the suggestion, that infection of house mice with canine *Hepatozoon* sp. is possible in single cases but their role in the respective infectious cycle remains circumstantial.

In our study, *Klossiella* sp. was detected in house mice inferring it as *Klossiella muris*, a commensal organism occurring in wildlife rodents as well as laboratory mice. The clinical relevance is described for laboratory mice (Yang and Grice 1964), but usually without clinical relevance under natural conditions (Rosenmann and Morrison 1975). The closest relative detected in GenBank based on 18S rRNA gene sequences obtained in this study is *Klossiella equi* detected in *Equus ferus caballus* (Leveille et al. 2019), the only known *Klossiella* nucleotide sequence until now. This finding is supported by organ tropism detected and morphology shown in our study that is typical for this species. The microscopic visualization of various infectious stages of *Klossiella* in our study was possible in the glomerular and tubular endothelium from kidneys of infected animals corresponding well with published data (Smith and Johnson 1902; Yang and Grice 1964; Elmadawy and Radwan 2011; Leveille et al. 2019; Camarinho et al. 2021). This infection resulted in interstitial inflammation only in single cases without any alterations in gross pathology, further confirming a naturally occurring infectious cycle. All *Klossiella muris* cases verified by pathology, resulted in positive results with our newly established *Klossiella*-specific qPCR (Adel2 assay). Detection by HTS without preamplification further excluded other infectious sources.

Samples from five house mice were positive in both apicomplexan assays (Supplementary Tables S1 and S2) suggesting double infections with *Hepatozoon* sp. and *Klossiella muris* Although PCR results were not verified for both protozoans via sequencing, it has to be mentioned that both qPCRs target different regions within the 18S rRNA gene. The Adel2 assay specific for *Klossiella* sp. in particular was designed to detect a genomic insert not present in *Hepatozoon* sp. sequences known so far. Co-infections with different parasites not only in vectors but also in their hosts can be classified as a naturally occurring phenomenon (Rizzoli et al. 2004; Broker 2012). Moreover, there is increasing evidence for co-infections of different agents, e.g., endoparasites and viruses or bacteria and viruses, or multiple co-infections (Telfer et al. 2010; Salvador et al. 2011; Guivier et al. 2014; Schmidt et al. 2014; Madrières et al. 2019; Jeske et al. 2021; Brila et al. 2022; Schlohsarczyk et al. 2023). Such co-infections might be important in several ways. On one hand, the infection with one infectious agent might enhance the susceptibility to get infected by another one or modify the outcome of disease. On the opposite, the infection with one infectious agent might decrease the susceptibility to get infected by another one. In addition, the immune status that is modulated by the microbiome might influence the susceptibility or outcome of an infection by a pathogen; or vice versa, the systemic infection with a pathogen may influence the gut microbiome in structure and functionality (Brila et al. 2022). Nevertheless, this study corroborates current knowledge that infections with multiple pathogens are a rather normal phenomenon. This has to be mentioned, when preventive measures follow prevalence-based risk analysis of possible reservoirs for single infectious diseases. Thus, a more comprehensive approach to screen for infectious agents in general is needed but might be challenging due to limitations in diagnostic capacities. Though further improvement of validated diagnostic approaches from a more general screening to more detailed verification could be an option.

In conclusion, we were able to sequence complete 18S rRNA sequences of *Klossiella muris* for the first time and of documenting its taxonomic characterization as well as naturally occurring life stages of this well-known commensal in mice. Furthermore, the diagnostic value of molecular assays is shown, especially in regions with unknown occurrence of pathogens. Molecular screening with at least genus-specific but sensitive diagnostic methods like qPCR complemented by pathology and sequence-based analysis enabled the verification of so far unknown pathogens in the respective region although sequence data were not available in all cases. From the preventive perspective, we were able to show the absence of medically relevant protozoans in small mammals inside military camps, but identified veterinary pathogens relevant for military service dogs. A consequence should be increased awareness and further in-detail training of dog handlers how to care for their dogs including the use of repellents against ticks. Nevertheless, the risk of zoonotic pathogen transmission between humans and animals including vectors (Benchaoui 2010) have to be considered in military camps. Besides the necessity of more comprehensive field studies covering different hosts and vectors to document naturally occurring life cycles, sensitive non-target diagnostic approaches can be helpful for the detection of taxa with hitherto unknown gene and genome sequences.

## Supplementary Information

Below is the link to the electronic supplementary material.

Supplementary file1 (DOCX 61405 KB)

Supplementary file2 (TIF 61374 KB)

## Data Availability

The nucleotide sequence data reported in this study are available in the GenBank under the NCBI accession numbers MT664753–MT664772.
