# Supplementary material for: Field survey and molecular characterization of apicomplexan parasites in small mammals from military camps in Afghanistan
Source: Parasitol Res. 2023 Mar 22;122(5):1199–211. doi: 10.1007/s00436-023-07820-8 (PMC10097762; doi:10.1007/s00436-023-07820-8)
Supplement: Supplementary file 1 — Supplementary file1 (DOCX 61405 KB) [file 436_2023_7820_MOESM1_ESM.docx]

**Appendix A. Supplementary data**

**Supplementary Table S1** Characterization of *Klossiella muris* (adeleorinid cluster 2-specific assay “Adel2”) qPCR-positive house mice (*Mus musculus*) from Mazar-e Sharif (MES), Kunduz (KDZ), and Feyzabad (FEY) with the respective accession numbers of a 650 bp segment from the 5’ end of the 18S rDNA small subunit or nearly complete 18S-rDNA sequences after high throughput-sequencing and histopathological findings.

| **ID** | **Sampling Site** | **Sampling Year** | **Kidney cq** Adel1 | **Kidney cq**Adel2 | **Spleen cq** Adel1 | **Spleen cq** Adel2 | **Liver cq** Adel1 | **Liver cq** Adel2 | **Accession Number** | **Histopathology** |
| --- | --- | --- | --- | --- | --- | --- | --- | --- | --- | --- |
| KS/11/1461 | MES | 2010 | - | 27.03 | - | 38.79 | nd | nd | MT664758 | Kidney: interstitial nephritis with intralesional *Klossiella* |
| KS/11/1463 | MES | 2009 | - | 39.91 | - | - | nd | nd | - | Lung: edema |
| **KS/11/1464** | **MES** | **2009** | **39.36** | **30.94** | **-** | **39.98** | **nd** | **nd** | **-** | Lung: edema |
| KS/11/1472 | MES | n/a | - | 28.97 | - | 46.00 | nd | nd | - | Lung: edema |
| KS/11/1479 | MES | 2009 | - | 25.45 | - | 36.25 | - | 34.3 | MT664760 | Kidney: interstitial nephritis |
| KS/11/1485 | MES | 2009 | - | 40.8 | nd | nd | nd | nd | - | Muscle: suppurative myositis |
| KS/11/1490 | FEY | 2009 | - | 22.12 | - | 37.17 | nd | nd | MT664761 | Kidney: interstitial nephritis with intralesional *Klossiella* |
| KS/11/1494 | MES | 2009 | - | 25.99 | - | - | - | 35.08 | MT664762 | Lung: edema |
| KS/11/1505 | MES | 2009 | - | 26.36 | - | 37.28 | nd | nd | MT664763 | No lesions |
| KS/11/1507 | MES | 2009 | - | 39.7 | - | - | nd | nd | - | Lung: edema |
| KS/11/1534 | MES | 2009 | - | 37.73 | - | nd | nd | nd | - | Liver: cestodiasis |
| KS/11/1547 | MES | 2009 | 38.45 | 40.9 | 41.36 | nd | nd | nd | - | Kidney: interstitial nephritis. lung: edema |
| KS/11/1607 | KDZ | 2009 | - | 38.73 | - | - | nd | nd | - | Autolysis |
| KS/11/1608 | KDZ | 2009 | - | 28.42 | - | 36.39 | nd | nd | - | Kidney: interstitial nephritis with intralesional Klossiella |
| KS/11/1616 | KDZ | 2009 | - | 37.46 | - | nd | nd | nd | - | Kidney: interstitial nephritis |
| KS/11/1691 | MES | 2010 | - | 26.33 | 42.98 | - | nd | nd | MT664769 | Kidney: interstitial nephritis with intralesional Klossiella |
| KS/11/1712 | MES | 2010 | 39.95 | 30.18 | 45.07 | - | nd | nd | - | Kidney: interstitial nephritis with intralesional Klossiella  Lung: edema |
| KS/11/1730 | MES | 2011 | - | 38.94 | nd | nd | nd | nd | - | Heart: myocarditis;  Liver: pericholangitis. fibrosis |
| KS/11/1741 | MES | 2011 | - | 40.79 | nd | nd | nd | nd | - | Kidney: interstitial nephritis with intralesional Klossiella |
| KS/11/1763 | MES | 2010 | 35.43 | 23.51 | 35.71 | 39.87 | 32.07 | 39.78 | MT664770 | Lung: edema. autolysis |
| KS/11/1769 | MES | n/a | - | 39.55 | nd | nd | nd | nd | - | Lung: edema |

n/a, unspecified sampling year; nd, samples for repeated testing not available; -, negative in the respective qPCR (Adel2) / PCR (BabR/BabF acc. to Inokuma et al. (2003)); Animals with double infection with *Hepatozoon* sp. (Adel1) and *Klossiella muris* (Adel2) are displayed in **bold**.

**Supplementary Table S2** Characterization of *Hepatozoon* spp. (adeleorinid cluster 1-specific assay “Adel1”) qPCR-positive small mammals (house mouse [*Mus musculus*]-**Mus**; Grey dwarf hamster [*Cricetulus migratorius*]-**CrM**; Lesser white-toothed Shrew [*Crocidura* cf. *suaveolens*]-**CrS**) and hard ticks [*Rhipicephalus* sp.-**RhS**]) from Mazar-e Sharif (MES), Kunduz (KDZ), and Feyzabad (FEY) with the respective accession numbers of a 780 bp segment from the 5’ end of the 18S rDNA small subunit and histopathological findings.

| **ID** | **Sampling Site** | **Species** | **Sampling Year** | **Kidney cq** Adel1 | **Kidney cq** Adel2 | **Spleen cq** Adel1 | **Spleen cq** Adel2 | **Liver qt** Adel1 | **Liver qt** Adel2 | **Accession No.** | **Histopathology / Remarks** |
| --- | --- | --- | --- | --- | --- | --- | --- | --- | --- | --- | --- |
| KS/11/1135 | MES | Mus | 2009 | - | - | 36.18 | - | nd | nd | - | NTA |
| KS/11/1136 | MES | Mus | 2009 | 25.74 | - | 20.00 | - | 24.39 | - | MT664753 | NTA |
| KS/11/1137 | MES | Mus | 2009 | - | - | 39.42 | - | nd | nd | - | Autolysis |
| KS/11/1138 | FEY | CrM | 2009 | - | - | 35.16 | - | nd | nd | - | Lung: edema |
| KS/11/1139 | MES | Mus | 2009 | - | - | 38.92 | - | nd | nd | - | Lung: edema |
| KS/11/1140 | MES | Mus | 2009 | - | - | 41.32 | - | nd | nd | - | Lung: intravascular microfilaria |
| KS/11/1141 | MES | Mus | 2009 | - | - | 39.92 | - | nd | nd | - | No lesions |
| KS/11/1142 | MES | Mus | 2009 | 39.05 | - | - | - | nd | nd | - | Kidney: interstitial nephritis |
| KS/11/1144 | MES | Mus | 2009 | 36.76 | - | 30.41 | - | 29.90 | - | - | No lesions |
| KS/11/1145 | MES | Mus | 2009 | - | - | 37.52 | - | nd | nd | - | No lesions |
| KS/11/1146 | MES | Mus | 2009 | - | - | 41.82 | - | nd | nd | - | Autolysis |
| KS/11/1147 | MES | Mus | 2009 | - | - | 37.23 | - | nd | nd | - | Autolysis |
| KS/11/1149 | MES | Mus | 2009 | 26.43 | - | 25.03 | - | 23.07 | - | MT664754 | Autolysis |
| KS/11/1150 | MES | Mus | 2009 | - | - | 40.27 | - | nd | nd | - | Lung: edema |
| KS/11/1151 | MES | Mus | 2009 | 40.13 | - | - | - | nd | nd | - | Autolysis |
| KS/11/1152 | MES | CrM | 2009 | - | - | 37.45 | - | nd | nd | - | Liver: pericholangitis Lung: edema |
| KS/11/1153 | MES | Mus | 2009 | - | - | 37.43 | - | nd | nd | - | No lesions |
| KS/11/1154 | MES | Mus | 2009 | - | - | 43.28 | - | nd | nd | - | Lung: edema |
| KS/11/1158 | MES | Mus | 2009 | - | - | 44.6 | - | nd | nd | - | Lung: suppurative bronchopneumonia |
| KS/11/1159 | MES | Mus | 2009 | 38.15 | - | - | - | nd | nd | - | Autolysis Lung: edema |
| KS/11/1160 | MES | Mus | 2009 | 35.54 | - | - | - | nd | nd | - | Autolysis |
| KS/11/1161 | MES | Mus | 2009 | 35.61 | - | - | - | nd | nd | - | No lesions |
| KS/11/1177 | MES | Mus | 2009 | 24.33 | - | 22.54 | - | 25.52 | - | MT664755 | Lung: edema Autolysis |

**Supplementary Figure S1.** **A**. Alignment of the 18S rDNA gene sequence of *Klossiella* sp. KS11/1479 and the partial sequence of *K. muris*. The insertion in the first sequence is marked. GC (blue graph) /AT (green line) content is given. **B**. Nucleotide sequence of the insertion of *Klossiella* sp. KS11/1479 (purines are rose-marked, pyrimidines are dark-blue marked). **C**. Dot plot analysis of the complete 18S rRNA gene sequence of *Klossiella* sp. KS11/1479 (horizontal axis) versus the insertion of this sequence (vertical axis). Small lines offset from the diagonal represent local repeat sequences (word size 10).
