# Supplementary figures and images for: Field survey and molecular characterization of apicomplexan parasites in small mammals from military camps in Afghanistan
Source: Parasitol Res. 2023 Mar 22;122(5):1199–211. doi: 10.1007/s00436-023-07820-8 (PMC10097762; doi:10.1007/s00436-023-07820-8)

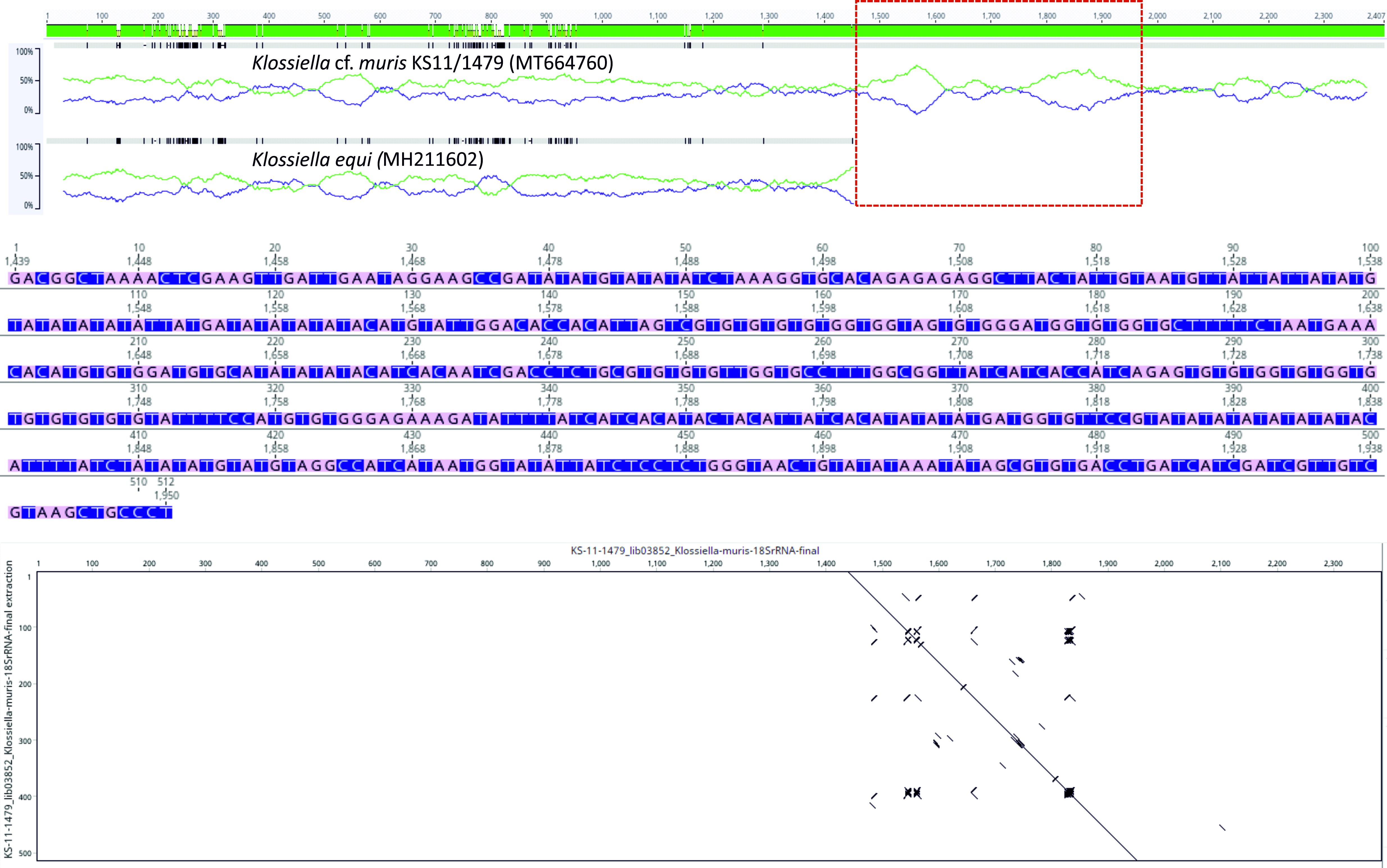

Supplement: Supplementary file 2 — Supplementary file2 (TIF 61374 KB) [file 436_2023_7820_MOESM2_ESM.tif]
